# A review of natural products as a source of next-generation drugs against apicomplexan parasites

**DOI:** 10.1038/s44259-025-00119-x

**Published:** 2025-06-06

**Authors:** Emma Y. Mao, Stephen W. Page, Brad E. Sleebs, Maria R. Gancheva, Danny W. Wilson

**Affiliations:** 1 Research Centre for Infectious Diseases, School of Biological Sciences, The University of Adelaide, Adelaide, SA 5005 Australia; 2 ARC Training Centre for Environmental and Agricultural Solutions to Antimicrobial Resistance (CEAStAR), St Lucia, Queensland Australia; 3 Institute for Photonics and Advanced Sensing (IPAS), University of Adelaide, Adelaide, 5005 SA Australia; 4 Neoculi Pty Ltd., Burwood, Victoria Australia; 5 Walter and Eliza Hall Institute of Medical Research, Parkville, Victoria 3052 Australia; 6 Department of Medical Biology, University of Melbourne, Parkville, 3010 Victoria Australia; 7 Burnet Institute, Melbourne, 3004 Victoria Australia

**Keywords:** Parasitology, Drug discovery

## Abstract

Despite the substantial global health and economic burden of apicomplexan parasites in humans and livestock, treatment options remain limited. Natural products have long played an important role in combating these diseases, offering diverse chemical structures and bioactive compounds. This review summarises past and present natural-product-based therapies for six economically significant apicomplexans and explores the potential of revisiting natural products as a source of next-generation treatments.

## Introduction

The phylum Apicomplexa represents a diverse group of intracellular, single-celled parasites, comprising more than 6000 known species^1^. These parasites are primarily distinguished by the presence of an apical complex, a specialised structure containing secretory organelles that facilitate host cell invasion^2,3^. Depending on the species, apicomplexan parasites can infect a wide range of vertebrate and invertebrate hosts, often involving complex, multi-host lifecycles that span various cell types and tissues^4^. Some of these parasites are prevalent globally and pose significant health and economic challenges as the causative agents of diseases in both humans and animals.

Current control and treatment options for apicomplexan parasites are limited. To date, there are only a small number of effective and commercially accessible vaccines for disease prevention^5^. The extensive use of pesticides to block transmission has led to widespread pesticide resistance across endemic areas^6–9^. Additionally, many of the clinically recommended and frontline drugs for treating apicomplexan parasites are hindered by their low efficacy^10–14^, toxic side effects^15^, and the emergence of parasite resistance mechanisms^16–22^. Therefore, there is an urgent need for new therapeutic options to combat the burden of apicomplexan parasites on public health and the global economy.

Natural products from plants, fungi, and bacteria have historically provided a rich source of new therapeutic agents, contributing to 66% of all small-molecule anti-infectives and 71% of approved cancer drugs between 1981 and 2019^23^. Several have had a profound impact on modern medicine, including the current frontline anti-malarial agent, artemisinin, whose discovery by Professor Youyou Tu earned her the 2015 Nobel Prize in Physiology or Medicine^24,25^. With >400,000 unique natural compounds curated to date^26^, and many more still to uncover from promising screens encompassing a diverse range of plants, fungi, bacteria, and even marine organisms^27–30^, there remains significant scope for natural products to be revisited as a source of next-generation drugs against apicomplexan parasites. This review summarises the most prominent natural products and their derivatives that have been clinically used to treat six apicomplexan genera that impose a significant global health and economic burden, these being *Plasmodium*, *Cryptosporidium*, *Toxoplasma*, *Eimeria*, *Babesia* and *Theileria*. Furthermore, we discuss the potential of natural products as future therapeutic agents given the deficiencies of current control strategies and the difficulty of developing an affordable and effective novel drug for use in all environments.

## Apicomplexan parasites of significant global health and economic burden

**Fig. 1:**
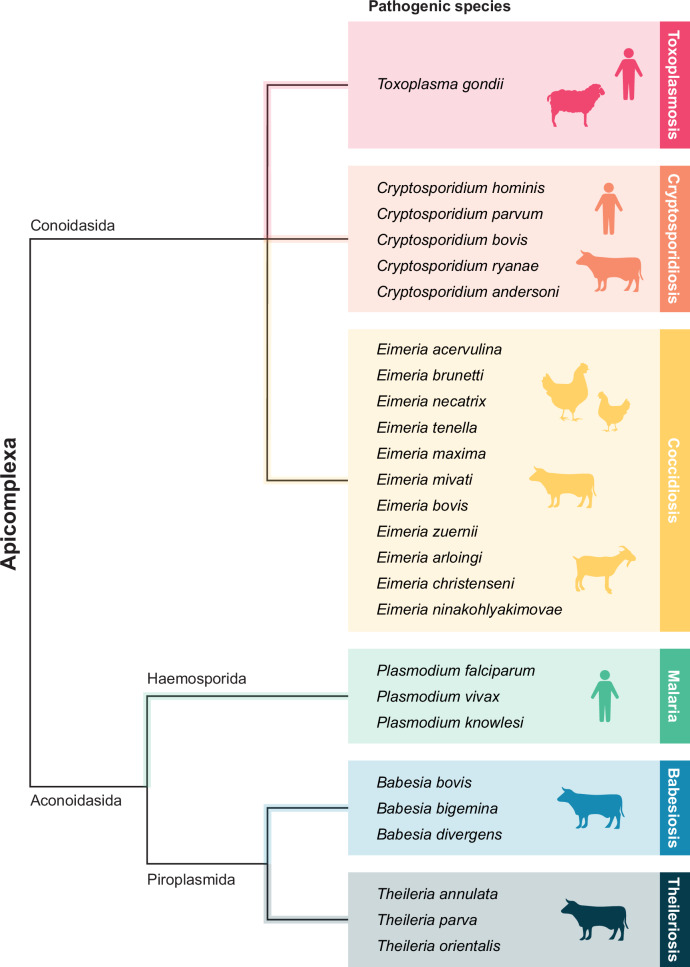
Phylogenetic tree of six prevalent and pathogenic genera of apicomplexan parasites that cause disease in humans and livestock. The Apicomplexa phylum comprises over 6000 species, capable of infecting a broad range of hosts. These six genera and species are the focus for this review as they have a disproportionately large health and economic impact on the represented hosts compared with other apicomplexans and/or have a more developed history of clinical drug use in humans and livestock.

Several parasites within the Apicomplexa phylum are responsible for severe diseases affecting either or both humans and animals, however, this review will focus on six key pathogens of notable economic significance that have approved drug treatments available (Fig. 1). While other apicomplexans also cause economically important levels of disease in livestock, such as *Neospora caninum*, *Sarcocystis* spp., *Isospora* spp., and *Besnoitia besnoiti*, specific treatments for these parasites are not typically available and treatments described in the literature are generally off-label uses of anti-parasitic drugs indicated for the treatment of other parasites.

Malaria, caused by *Plasmodium* spp., remains one of the most debilitating apicomplexan diseases in humans, affecting over 200 million people each year, leading to more than half a million fatalities^31^. Approximately 95% of all deaths occur in sub-Saharan Africa, of which around 80% are among children under the age of five^31^. Of the five human-infecting *Plasmodium* spp., *P. falciparum* stands as the deadliest and most prevalent in the African region, with *P. vivax* and *P. knowlesi* progressively emerging as substantial threats in the Americas and Southeast Asia^31^.

Cryptosporidiosis, caused by *Cryptosporidium* spp., is a leading cause of severe diarrhoeal disease in children, responsible for over 200,000 deaths annually in South Asia and sub-Saharan Africa alone^32^. These parasites are transmitted through the ingestion of infective oocysts that are highly resistant in the environment to chlorine, among other conventional disinfectants, rendering them a major threat in waterborne outbreaks^33^. Furthermore, up to one third of the global population is chronically infected with *Toxoplasma gondii*, the causative agent of toxoplasmosis^34^. Although considered asymptomatic in healthy individuals, *T. gondii* can cause severe complications in immunocompromised patients and pregnant women, including encephalitis, ocular damage, congenital defects and miscarriage^35–37^. In addition, *T. gondii* infections have been associated with an increased risk of chronic physical and mental health problems such as heart disease and schizophrenia^38–41^, suggesting that the broader health impacts of this widespread parasite could be much greater than indicated by acute infection.

The economic impact of apicomplexan diseases extends beyond human health to livestock, with several animals raised for food production facing constant threats from parasitic infection. Toxoplasmosis, for example, has long been associated with increased abortion rates in sheep^42–44^, although estimating its full economic impact has been challenging due to the complex factors influencing neonatal mortality^45^. Coccidiosis, caused by *Eimeria* species, and cryptosporidiosis, are well-known gastrointestinal illnesses of cattle, sheep, goats, and poultry, leading to diarrhoea, reduced growth rates, and, in severe cases, death^45,46^. Recent estimates indicate that coccidiosis alone costs the commercial poultry industry more than £10 billion annually^47^, while cryptosporidiosis can incur losses of up to €60 per affected calf on dairy farms in Europe, where approximately 11 million calves are raised for milk production each year^48^. Additionally, babesiosis and theileriosis, caused by *Babesia* and *Theileria* species, respectively, also contribute to significant livestock losses in cattle, costing industries over US $300 million in annual losses alongside other tick-borne diseases due to decreased dairy and meat production^49^. Many of these apicomplexan diseases disproportionately affect populations in low- and middle-income regions, further imposing a heavy financial burden on individuals, families and health systems^50,51^.

## Current therapeutic avenues for apicomplexan parasites

The control and eradication of apicomplexan pathogens have proven challenging, partly due to the complex, multi-host lifecycles of these parasites and the lack of readily available, and economically accessible resources in endemic areas. Currently, the only commercially approved vaccines for apicomplexans in humans are two recombinant subunit vaccines for malaria: RTS,S/AS01 and R21/Matrix-M^52,53^. For use in livestock, live attenuated vaccines are available for some apicomplexans in endemic areas, such as Toxovax® for *T. gondii* in sheep^54^, the Combavac 3 in 1 vaccine for *Babesia* in cattle^55^, and several commercial *Eimeria* vaccines^56^. Additionally, the recombinant antigen vaccine, Bovilis Cryptium, has also received United Kingdom Veterinary Medicines Directorate (VMD) approval in the last year for use in protecting newborn calves against *C. parvum* in pregnant cows^57^. However, concerns remain regarding the long-term efficacy of malaria vaccines^52,53^, and many livestock vaccines are geographically restricted for commercial use, leaving a significant proportion of endemic countries unprotected. For example, the Combavac 3 in 1 vaccine is currently only approved for use in Australia^56^, while Toxovax® is available in New Zealand and some parts of Europe^54^. Moreover, logistical challenges such as the short shelf-life of Toxovax® (less than 10 days) further highlight the difficulties of vaccine development for apicomplexans.

For vector-borne parasites like *Plasmodium*, *Babesia* and *Theileria*, chemical pesticides such as organophosphates and pyrethroids have been widely used to curb transmission, though their effectiveness is increasingly compromised by the emergence of insecticide and acaricide resistance^6–9^. For parasites that are spread through environmental contamination, such as *T. gondii*, *Cryptosporidium*, and *Eimeria*, meticulous hygiene and biosecurity measures are among the most important methods for disease prevention^34,58–60^.

Clinically approved drug treatments are available for disease management in both humans and livestock, however, treatment options remain limited for the majority of apicomplexans (Table 1). The World Health Organisation (WHO) currently recommends artemisinin-based combination therapies (ACTs) as the gold standard for malaria treatment^61^. ACTs combine a short-acting artemisinin-based anti-malarial with a longer-acting partner drug, such as lumefantrine, amodiaquine and mefloquine, to minimise the selective pressure for resistance development^61–64^. For the treatment of cryptosporidiosis, nitazoxanide is currently the only approved drug for human use, however, it is ineffective in those with weakened immune systems, who are at a greater risk of fatal disease^10,11^.

**Table 1:** Current frontline treatments for apicomplexans in humans and livestock

Disease (Parasite)	Host	Recommended treatment	Mechanism of action	Usage notes	References
Malaria (*Plasmodium* spp.)	Humans	Artemisinin combination therapy (ACT)	Primary artemisinin-based drugs produce toxic free radicals^92,93^.	Oral treatment for uncomplicated malaria^78^.	Adebayo et al. 2020^92^ Tilley et al. 2016^93^ WHO, 2023^78^
		Artesunate		Intravenous injection for severe malaria^78^.	
Cryptosporidiosis (*Cryptosporidium parvum*)	Humans	Nitazoxanide	Inhibits electron transport via pyruvate oxidoreductases in other microorganisms^208^.	Oral treatment for patients older than 12 months^209^.	Hoffman et al. 2007^208^ Ashigbie et al. 2021^209^
	Calves	Halofuginone	Inhibits prolyl-tRNA synthetase of malaria parasites^210^.	Oral treatment reduces oocyst shedding and manage diarrhoeal symptoms^70,211^.	Jain et al. 2014^210^ APVMA, 2007^211^
		Paromomycin	Inhibits protein synthesis of bacterial ribosomes^212^.		De Stasio et al. 1989^212^ Riviere et al. 2018^70^
Toxoplasmosis (*Toxoplasma gondii*)	Humans	Pyrimethamine plus sulfadiazine	Inhibits folate synthesis^213,214^.	Oral treatment for all presentations of toxoplasmosis and for pregnant women infected after 18 weeks of gestation^102^.	Ferone et al. 1969^213^ Triglia et al. 1997^214^ Goldstein et al. 2008^102^
		Spiramycin	Inhibits apicoplast protein synthesis^95^.	Oral treatment for pregnant women whose infections are acquired before 18 weeks gestation, where infection of foetus is not suspected^102^.	Pfefferkorn et al. 1994^95^ Goldstein et al. 2008^102^
	Livestock	None registered	–	–	Batey et al. 2024^75^
Coccidiosis (*Eimeria* spp.)	Poultry Cattle Sheep Goats	Polyether ionophores	Disrupts cation homeostasis^110,111^.	Typically used in rotation programs as prophylactics (polyether ionophores, diclazuril, nicarbazin) or treatments (amprolium, sulfonamides) added to feed or drinking water to reduce disease severity^215^.	Smith et al. 1981^110^ Smith & Galloway, 1983^111^ James, S. 1980^216^ Ferone et al. 1969^213^ Triglia et al. 1997^214^ Noack et al. 2019^215^
		Diclazuril	Unknown		
		Nicarbazin	Unknown		
		Amprolium	Inhibits thiamine synthesis^216^.		
		Sulfonamides	Inhibits folate synthesis^213,214^.		
Babesiosis (*Babesia bovis, B. bigemina, B. divergens*)	Cattle	Imidocarb	Disrupts DNA synthesis and parasite replication^217–219^.	Subcutaneous injection largely used to prevent development of clinical disease in healthy cattle entering tick-endemic areas^220,221^.	Ariyibi et al. 2001^217^ Patrick et al. 1997^218^ Pilch et al. 1995^219^ Silva et al. 2020^220^ Kuttler et al. 1975^221^
Theileriosis (*Theileria parva, T. annulata*)	Cattle	Buparvaquone	Structural analogues inhibit mitochondrial electron transport^222,223^.	Intramuscular injection registered in around 20 countries for the treatment of East Coast fever and tropical theileriosis^157^.	Fry & Pudney, 1992^222^ Srivastava et al. 1997^223^ Carter, P. 2011^157^

For human infections with toxoplasmosis, a combination of pyrimethamine and sulfadiazine is the most widely used therapy^15^. However, this combination is often poorly tolerated, with one study reporting adverse side effects in up to 60% of toxoplasma encephalitis patients, of which 45% required treatment discontinuation^15^. Unfortunately, few alternatives are available for those who cannot tolerate standard treatment. Repurposed antibiotics such as spiramycin, and to a lesser extent clindamycin and azithromycin, have been used or trialled as alternatives to treat active infections, but there is limited evidence supporting their efficacy^12–14,65^.

In veterinary medicine, drug treatments have demonstrated varying effectiveness against apicomplexan parasites. For cryptosporidiosis in calves, halofuginone and paromomycin are the two primary treatments used to reduce oocyst shedding and disease severity^66–69^, whereas imidocarb is the drug of choice for bovine babesiosis^70,71^, and buparvaquone for bovine theileriosis^70,72^. Coccidiosis in chickens is managed with a range of natural polyether ionophores and synthetic drugs, with the choice of drug largely based on where in the lifecycle the drug is most effective and previous anti-parasitic drug use for the flock. Most drug use for coccidiosis in chickens is for prevention, including the ionophores, diclazuril and nicarbazin, whereas amprolium and antifolates are primarily used for treatment^70^. Many of these anti-coccidials are also recommended for clinical treatment of coccidiosis in pigs caused by the closely related *Isospora suis*^73,74^. In the case of toxoplasmosis, there are currently no anti-toxoplasma treatments registered for use in livestock^75^. Many existing drugs, including spiramycin, pyrimethamine, sulfonamides, polyether ionophores, and other anti-coccidials, have been trialled against *T. gondii* in sheep and the closely related *N. caninum* in cattle, however, no feasible treatment regimen has emerged for clinical use against ovine toxoplasmosis or bovine neosporosis^76^.

## Historical importance of natural products in the treatment of apicomplexans

**Fig. 2:**
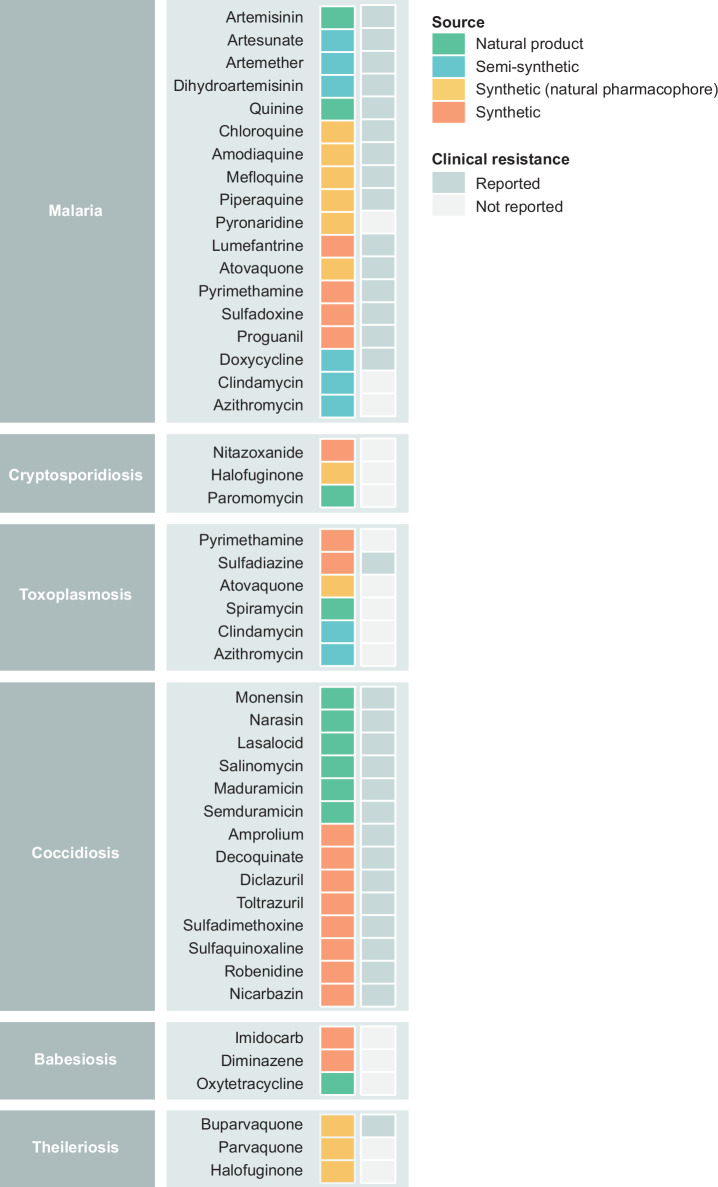
Most widely used drugs in human and veterinary medicine for the treatment of apicomplexan infections. Around 39% are natural products or chemically modified derivatives (semi-synthetic), and a further 22% are synthetic compounds inspired by natural pharmacophores. Clinical resistance has emerged to the majority of these drugs, leaving few alternatives with strong therapeutic potential available.

Many pharmaceuticals on the market today have been developed from natural products or their derivatives^77^. Among the current most widely used drugs in clinical settings for apicomplexan infections in humans and livestock (Fig. 2)^61,70,78^, around 39% are natural products or their semi-synthetic derivatives, and a further 22% are synthetic compounds inspired by naturally occurring pharmacophores. Tables 2 and 3 provide a summary of these natural products and showcase the range of derivatives that have arisen from natural product scaffolds, either made synthetically, or semi-synthetically by chemical modification of existing natural products. In the following, we summarise the naturally derived drugs that have either historically been used, or are currently in use, to treat apicomplexan infections.

**Table 2:** Natural products clinically used for treating infections with apicomplexan parasites

Disease (Parasite)	Host	Drug class	Compound	Source	Mechanism of action	References
Malaria (*Plasmodium* spp.)	Humans	Sesquiterpene lactone	Artemisinin	*Artemisia annua*	Production of toxic free radicals^92,93^.	Adebayo et al. 2020^92^ Tilley et al. 2016^93^
		Cinchona alkaloid	Quinine	*Cinchona* tree bark	Production of toxic haem products^87^.	Sullivan et al. 1998^87^
Cryptosporidiosis (*Cryptosporidium parvum*)	Calves	Aminoglycoside antibiotic	Paromomycin	*Streptomyces* spp.	Inhibits protein synthesis of bacterial ribosomes^212^.	De Stasio et al. 1989^212^ Armitage et al. 1992^122^
Toxoplasmosis (*Toxoplasma gondii*)	Humans	Macrolide antibiotics	Spiramycin	*Streptomyces ambofaciens*	Inhibits apicoplast protein synthesis^95^.	Pfefferkorn et al. 1994^95^
Coccidiosis (*Eimeria* spp.)	Poultry Cattle Sheep Goats	Polyether ionophores	Monensin Narasin Lasalocid Salinomycin	*Streptomyces* spp.	Disrupts cation homeostasis^110^.	Smith, Galloway & White, 1981^110^
			Maduramicin Semduramicin	*Actinomadura* spp.		
Babesiosis (*Babesia bovis, B. bigemina, B. divergens*)	Cattle	Tetracycline Antibiotic	Oxytetracycline	*Streptomyces rimosus*	Structural analogues inhibit apicoplast protein synthesis^129^.	Dahl et al. 2006^129^

**Table 3:** Semi-synthetic and synthetic derivatives of clinically used natural products

Disease (Parasite)	Host	Compound	Origin	Derived from	Mechanism of action	References
Malaria (*Plasmodium* spp.)	Humans	Artesunate Artemether Dihydro-artemisinin	Semi-synthetic	Artemisinin	Production of toxic free radicals^92,93^.	Adebayo et al. 2020^92^ Tilley et al. 2016^93^
		Chloroquine Amodiaquine Mefloquine Piperaquine Pyronaridine	Synthetic	Quinine	Production of toxic haem products (mefloquine is also thought to have a cytosolic mode of action)^85,87,224,225^.	Fitch, C. 1998^85^ Sullivan et al. 1998^87^ Wong et al. 2017^224^ Sheridan et al. 2018^225^
		Atovaquone	Synthetic	Lapachol	Inhibits mitochondrial electron transport via the cytochrome bc_1_ complex^222,223^.	Fry & Pudney 1992^222^ Srivastava et al. 1997^223^
		Doxycycline	Semi-synthetic	Tetracycline	Inhibits apicoplast protein synthesis^131^.	Dahl et al. 2007^131^
		Clindamycin	Semi-synthetic	Lincosamide		
		Azithromycin	Semi-synthetic	Macrolide		
Cryptosporidiosis (*Cryptosporidium parvum*)	Calves	Halofuginone	Synthetic	Febrifugine	Inhibits prolyl-tRNA synthetase of malaria parasites^210^.	Jain et al. 2014^210^ Villacorta et al. 1991^226^
Toxoplasmosis (*Toxoplasma gondii*)	Humans	Atovaquone	Synthetic	Lapachol	Inhibits mitochondrial electron transport via the cytochrome bc_1_ complex^222,223^.	Fry & Pudney, 1992^222^ Srivastava et al. 1997^223^
		Clindamycin	Semi-synthetic	Lincosamide	Inhibits apicoplast protein synthesis^131^.	Dahl et al. 2007^131^
		Azithromycin	Semi-synthetic	Macrolide		
Coccidiosis (*Eimeria* spp.)	Chickens	Halofuginone	Synthetic	Febrifugine	Inhibits prolyl-tRNA synthetase of malaria parasites^210^.	Jain et al. 2014^210^ Zhang et al. 2012^162^
Theileriosis (*Theileria parva, T. annulata*)	Cattle	Buparvaquone Parvaquone	Synthetic	Lapachol	Structural analogues inhibit mitochondrial electron transport^222,223^.	Fry & Pudney, 1992^222^ Srivastava et al. 1997^223^
		Halofuginone	Synthetic	Febrifugine	Inhibits prolyl-tRNA synthetase of malaria parasites^210^.	Jain et al. 2014^210^ Kiltz & Humke, 1986^227^

### Quinine

Quinine, first isolated in 1820 from the bark of the Peruvian *Cinchona* tree, had been used medicinally to treat a variety of fevers in South America for centuries before introduction to Europe in the 1600s where it was found by analogy to be useful in the treatment of intermittent fevers associated with malaria^62^. Quinine remained the frontline anti-malarial until the 1940s, by which point access to more effective synthetic drugs was facilitated by the US Army’s malaria drug discovery programs^62^. The most important of these was chloroquine, a quinoline-containing compound first described in 1938^79,80^ that became heavily used in the 1940s^81^, and is still recommended in areas with chloroquine-susceptible infections^61^. Several other synthetic derivatives discovered after chloroquine, such as amodiaquine, mefloquine, piperaquine and pyronaridine, are incorporated as partner drugs in ACTs^81–84^. Although the exact mechanism of action for many quinoline-based drugs remains an active area of research, it is thought that in part, their efficacy is via inhibiting the polymerisation of toxic haem released during the parasite’s digestion of haemoglobin^85–87^.

### Artemisinin

Artemisinin was discovered in 1972 as the active anti-malarial component of *Artemisia annua*, a herbal plant traditionally used to treat fevers and chills during malaria infections^88^. Unlike previous anti-malarials, artemisinin was shown to rapidly clear malaria parasites within hours of administration, however, its short half-life has limited its use as an oral monotherapy^89,90^. Subsequent modifications to artemisinin led to the development of analogues with enhanced metabolic stability and efficacy, including dihydroartemisinin, artemether, and artesunate, which are now clinically used in ACTs for uncomplicated malaria and as intravenous monotherapies for severe malaria^61,91^. The unique structure of the artemisinins offers a distinct mechanism of action against malaria parasites, differentiating them from previous quinine-derived and synthetic drugs. It is now widely accepted that the bioactivation of artemisinin from iron-mediated cleavage of the endoperoxide bridge generates free oxygen radicals that promiscuously alkylate essential proteins and biomolecules, ultimately leading to parasite death^92,93^.

### Spiramycin

Spiramycin is a macrolide antibiotic originally isolated from *Streptomyces ambofaciens* in 1954^94^. In apicomplexan parasites, spiramycin is thought to inhibit protein synthesis in the bacterial-like ribosomes of an essential plastid organelle called the apicoplast^95^. One of the first studies to demonstrate the anti-*Toxoplasma* potential of spiramycin in humans was conducted in 1961 for ocular toxoplasmosis^96^, though its primary clinical use has since been for preventing congenital toxoplasmosis, to avoid potential teratogenic risks associated with the standard pyrimethamine-sulfadiazine combination therapy^97^. Studies in pregnant women have shown that spiramycin can reduce congenital transmission of *T. gondii* to the foetus, potentially by accumulating in the placenta^98–100^. However, since transfer of spiramycin across the placental barrier is considered inefficient^101^, spiramycin is typically withdrawn once foetal transmission is confirmed and replaced with the conventional pyrimethamine and sulfadiazine regimen^102^. Furthermore, the drug is not suitable for treating toxoplasma encephalitis, as it cannot cross the blood-brain barrier^103,104^.

### Polyether ionophores

Polyether ionophores such as monensin, narasin, salinomycin and lasalocid are secondary metabolites recovered from the fermentation of several *Streptomyces* species^105,106^, and are the predominant anti-coccidials used by the broiler industry since the 1970s to control and reduce the prevalence of coccidiosis in poultry^107^ and other livestock species. Studies suggest that these drugs inhibit host cell invasion and intracellular parasite development^108,109^, potentially by disrupting cation homeostasis^110,111^. The use of these natural products is now controlled by many poultry producers in the United States and subject to greater regulation in the European Union, partly due to the anti-bacterial activity of the ionophores and concerns about the potential spread of antimicrobial resistance co-selected to antibiotics of public health importance in human food products^112,113^. Public health risks associated with the use of the ionophores have been described as low^114,115^ but data is incomplete and ongoing surveillance of antimicrobial resistance and emergence of new risks is advocated^116,117^.

### Paromomycin

Paromomycin is an aminoglycoside antibiotic isolated from *Streptomyces rimosus* forma *paromomycinus*^118^, and other species, with broad-spectrum activity against a number of bacteria and intestinal parasites^69^. In bacteria, the drug binds to ribosomal RNA and inhibits translocation during protein synthesis^119^, whereas in the kinetoplast *Leishmania*, it has been suggested to interfere with parasite mitochondrial activity^120^. Paromomycin first demonstrated potential against *Cryptosporidium parvum* in the early 1990s, however, its poor bioavailability when taken orally has limited its application for human use^121–123^. The drug was later evaluated in 1993 for efficacy in dairy calves, which showed that as a prophylactic, it was capable of reducing oocyst shedding and the severity of diarrhoea^69^. While studies have suggested that paromomycin inhibits the intracellular growth of *C. parvum*, the mechanism by which this occurs remains unclear^121–123^.

### Oxytetracycline

Oxytetracycline (OTC), produced by *Streptomyces rimosus*, is a broad-spectrum antibiotic used widely in veterinary medicine since the early 1950s to treat bacterial and protozoal infections^70,124,125^. For *Theileria* infections in cattle, OTC has been used in conjunction with immunisation of a low infective dose of live parasites to reduce disease severity without inhibiting the development of natural immunity^126^. OTC was later shown to be effective against infection with *Babesia* in cattle, with high doses capable of completely inhibiting parasite replication and lower doses controlling parasitaemia while maintaining antibody responses^127,128^. The mechanism of action of OTC in these parasites has not been described, though it is likely similar to related tetracyclines like doxycycline, a suppressive anti-malarial prophylactic, which works against *Plasmodium* species by inhibiting protein synthesis in the apicoplast, leading to a subsequent loss of apicoplast function and a delayed, but potent, anti-malarial effect^129^.

### Semi-synthetic antibiotics

Several antibiotics repurposed for use against apicomplexan parasites are synthetically modified analogues of natural products that target the translation of apicoplast proteins^129–131^. Doxycycline, for example, was chemically modified from OTC, and is now recommended for malaria prophylaxis by travellers entering malaria-endemic areas or occasionally as an alternative partner drug in ACTs where traditional combinations fail^61^. Clindamycin, derived from the microbial metabolite, lincomycin, is primarily administered as a safe and effective anti-malarial in the first trimester of pregnancy, or where traditional ACTs are unavailable^61,132^. The drug also acts as an alternative partner drug in combination therapies for toxoplasmosis patients who are intolerant to sulfonamides and pyrimethamine^133,134^, however, its efficacy against *T. gondii* is still controversial^135^.

Additionally, azithromycin, synthetically modified from the macrolide erythromycin, has been trialled as a treatment for cerebral toxoplasmosis in immunocompromised patients^136^, and as both a prophylactic and partner drug in malaria combination therapies^137–140^. However, there has been limited progress into clinical implementation as azithromycin’s efficacy in its current form is suboptimal compared with other clinically useful drugs^140–142^. Several studies have shown that further modification of azithromycin is capable of significantly improving the drug’s activity, making it of similar potency to fast-acting anti-malarials like chloroquine and artemisinin in vitro^143–146^.

### Synthetic compounds with naturally occurring pharmacophores

Several natural products with demonstrated activity against apicomplexan parasites have not been pursued clinically for the treatment of human and animal diseases due to drug toxicity, or the emergence of new and more effective synthetic derivatives. For instance, many widely used drugs in human and veterinary medicine, such as chloroquine, atovaquone, buparvaquone and halofuginone, are synthetically made compounds that mimic key components of their natural counterparts.

Lapachol, first extracted from the bark of *Tabebuia avellanedae* trees in 1882, was one such natural product^147^. Like many other naphthoquinones, lapachol acts as an inhibitor of the mitochondrial electron transport chain and has shown efficacy in suppressing malaria in animals^148,149^. The identification of naphthoquinones as potential anti-malarial agents led to the discovery of more potent analogues like atovaquone^150–152^. Atovaquone was initially approved by the United States Food and Drug Administration (FDA) in 1995 as a monotherapy for malaria, but due to high rates of treatment failure driven by drug resistance, it was later combined with proguanil for malaria prophylaxis to reduce future resistance selection^153,154^. In addition to its anti-malarial activity, atovaquone has shown efficacy against other apicomplexan parasites, such as *T. gondii*^155^ and the zoonotic *Babesia microti*^156^. Parvaquone and buparvaquone also emerged as effective synthetic derivatives of lapachol and have been used to treat *Theileria* infections in cattle^70^. Buparvaquone has previously been shown to be more than 20-fold more active than parvaquone against *T. parva* and *T. annulata*, making it the preferred drug for treatment of African East Coast fever and tropical theileriosis in around 20 countries^157^.

Febrifugine, first isolated from the Chinese herbal plant, *Dichroa febrifuga*, is another natural product that demonstrated potent activity against *Plasmodium* spp. but was unable to be further developed for human use due to its low margin of safety and presence of unacceptable adverse effects^158–160^. Synthetic derivatives of febrifugine later yielded analogues like halofuginone, which was comparatively less toxic and developed commercially for veterinary use as a therapeutic and preventative agent for cryptosporidiosis in calves and coccidiosis in poultry^160^. Several studies reported that halofuginone reduces oocyst shedding of *Cryptosporidium* and *Eimeria* in cattle and chickens, significantly enhancing body weight gains when compared with untreated infected animals^67,161,162^. In *Plasmodium* spp., halofuginone is known to inhibit prolyl-tRNA synthetase^163^, and it is expected to have a similar effect in other apicomplexans^164^.

## Current progress and challenges in drug discovery and development for apicomplexan parasites

While significant progress has been made to address the global health and economic burden of diseases caused by apicomplexans through both synthetic and natural therapeutic agents, these parasites continue to have a severe impact on human and animal health worldwide. The majority of the drugs currently used for treating these diseases are decades old, with drug resistance emerging as a major cause of treatment failure (Fig. 2). Resistance has now developed to all major classes of anti-malarials, including the current frontline ACTs^16–19^. The widespread reliance on anti-coccidials for prophylaxis and treatment in poultry farming has contributed to the selection of *Eimeria* strains resistant to all synthetic and natural anti-coccidials^20–22^. Although less prevalent, treatment failures have now been reported to buparvaquone in animals infected with *T. annulata*^165^ and mutations associated with sulfonamide resistance have been identified in clinical isolates of *T. gondii*^166^. Few alternatives with strong therapeutic potential are available. Currently, many of the natural products with activity against apicomplexan parasites target anti-microbial protein synthesis, and are typically used as suppressive preventative treatments rather than curative treatments due to their slow-acting mechanism of killing^131^. Therefore, there is an urgent need for next-generation drugs that are not only highly effective, preferably against multiple stages of the parasite’s lifecycle, but also selective with low likelihood of resistance selection.

Despite the ongoing global impact of apicomplexan parasites, only 11 new anti-parasitic drugs with apicomplexan activity have been approved for human use since 1981, and all are principally active against malaria^23^. In terms of anti-parasitic drugs for veterinary use, even fewer have become available due to cost-effectiveness being the over-riding consideration for development and use. Livestock diseases typically attract more attention for drug development when they pose a significant threat to profitable production and offer clear economic benefits following treatment^167^. For example, *Eimeria*, a major threat to intensive poultry production worldwide, has prompted the development of several anti-coccidial drugs, both natural and synthetic, to meet the growing demands in the poultry industry^168^, though only one new class (triazines) has been introduced in almost 40 years^169^. In contrast, diseases like babesiosis and theileriosis primarily affect livestock in tropical and subtropical regions, including parts of Africa and Asia where small-scale farming systems lack the financial resources to afford expensive treatments, regardless of their efficacy^51^. Therefore, treatment options for babesiosis and theileriosis are fewer and the incentive for dedicated drug development programs is less favourable. Other diseases like toxoplasmosis have often been overlooked as their economic impact is harder to quantify^167,170^, and infection in livestock is typically not detected until after abortions have occurred, by which point treatment offers little to no economic or animal health benefit^76^.

In recent years, partnerships with not-for-profit organisations (e.g. the Medicines for Malaria Venture, Drugs for Neglected Diseases), funding agencies, (e.g. Wellcome Trust, Bill and Melinda Gates Foundation), industries, and certain pharmaceutical companies, have made considerable contributions to the discovery of new drug candidates^171^. Several of these new drugs have reached late-stage clinical trials, including EDI048 for cryptosporidiosis^172^, and cipargamin, SJ733 and DSM265 for malaria^173^. Of note, cipargamin, is a synthetic compound featuring an indole group common to many natural products^174^, originally identified as NITD609 from a large library of pure natural products and synthetic compounds with natural product-derived structural features^175^. These spiroindolones now represent a new class of anti-malarials that inhibit the *P. falciparum* ATP4ase^175,176^. While most of these drugs are primarily intended for human use, some may have potential to be repurposed for the treatment of other apicomplexan infections in livestock due to common drug targets. Especially for neglected livestock diseases, drug repurposing may be an attractive and economical option, however, this could contribute to the selection and spread of drug resistance, which is of greatest public health importance with zoonotic apicomplexans.

## Potential for development of natural products as next-generation drugs against apicomplexans

Given the historical importance of natural products for treating apicomplexan parasites, harnessing these compounds as an avenue for future drug development may provide a promising opportunity to discover novel chemical entities with therapeutic potential. Compared to conventional synthetic molecules, natural products are known to offer a broader range of chemical diversity and structural complexity^177,178^, typically featuring molecular scaffolds that have evolved to interact with proteins, enzymes and other biological molecules^177,178^. Many natural products and their semi-synthetic derivatives currently used to treat apicomplexan infections target protein synthesis in the parasite’s apicoplast and have been repurposed from their original use as anti-microbials. However, this approach carries the risk of contributing to resistance in other organisms and the environment, making it a less sustainable strategy for disease control. Therefore, screening for and identifying natural products with apicomplexan-specific mechanisms of action would offer a more promising alternative.

Several promising screens have been conducted with large compound and extract libraries sourced from plants, fungi, bacteria, and marine organisms against *Plasmodium*^27,30,175,179,180^, *Cryptosporidium*^28,181,182^, *Toxoplasma*^183–186^, *Eimeria*^187–190^, and *Babesia*^191–193^ (Table 4). These studies have led to the identification of a number of bioactive compounds with low cytotoxicity, some of which have known activity in other systems, whereas others are entirely novel. With over 400,000 unique natural compounds already curated to date^26^, there remains significant scope to build upon existing screens against apicomplexans and repurpose promising natural products. Most notably, these studies highlight the incredible diversity and untapped potential of bioactive compounds from a wide array of natural sources yet to be discovered. This offers an exciting pathway forward for drug discovery and development, not only against the six genera covered in detail in this review, but also against other apicomplexans like *Neospora*^76^, *Sarcocystis*^194^, and *Besnoitia*^195^, which lack effective treatment options and pose significant health and economic challenges in livestock.

**Table 4:** Natural products with promising in vitro activity that have arisen from screening studies against apicomplexan parasites

Parasite	Natural product	Chemical class	Source	In vitro IC_50_ (µM)^a^	Cytotoxicity (SI)^b^	References
*P. falciparum*	Bebrycin A	Terpenes	Marine organisms	1.08	20	Wright et al. 2021^27^
	Nitenin			0.29	63	
	Cladosporin	Isocoumarin	Fungi	0.05	213	Hoepfner et al. 2012^29^
	Alstonine	Alkaloids	Plants	0.18	>1111	Arnold et al. 2021^179^
	Himbeline			0.77	>144	
	Berberine chloride			0.03	ND^c^	Nonaka et al. 2018^180^
	Coptisine chloride			0.04	ND	
	Palmatine chloride			0.04	ND	
	Dehydrocorydaline nitrate			0.04	ND	
*C. parvum*	Leiodolide A	Macrolide	Marine organisms	0.10	12 – 45	Relat et al. 2022^28^
	Cedrelone	Limonoid	Plants	0.27	13.4	Jin et al. 2019^181^
	Deoxysappanone B 7,4’-dimethyl ether	Flavonoids		0.73	88.9	
	Baicalein			0.98	102	
	Alisol-A	Terpenes		0.12	484	Kabir et al. 2022^182^
	Alisol-B			0.08	466	
	Atropine sulfate^d^	Alkaloid		0.03	70	
	Bufotalin^d^	Lactone		0.06	172	
*T. gondii*	Kavain	Pyranone	Plants	2.61	130	Adeyemi et al. 2018^183^
	Emodin	Quinone		0.15	120	
	Isosakuranetin	Flavonoid		0.70	161	
	Maritimein	Phenol		0.07	20	
	Efrapeptin analogues	Peptaibol	Fungi	0.01 – 0.06	>20	Jiang et al. 2024^184^
	Verticillin analogues	Alkaloid		0.01 – 0.04	26 – 25	
	Apicidin analogues	Cyclic Tetrapeptides		0.03 – 0.06	>633	
	1-Alaninechlamydocin			0.03	>697	
	Xanthoquinodin analogues	Anthraquinone		0.06 – 0.13	115 – 218	
	Fumagillin	Terpenoid		0.06	>340	
*B. bovis* *B. microti*	Rottlerin	Polyphenolic	Plants	5.45	5	Li et al. 2021^192^
	Narasin	Polyether ionophores	Bacteria	1.86	46	
	Lasalocid			3.56	3	
*B. gibsoni*	Lycorine	Alkaloids	Plants	0.78	1639	Ji et al. 2021 ^193^
	Vincristine sulfate			0.64	81	
	Emetine			0.25	877	
	Harringtonine			0.023	49658	
	Cephaeline			0.11	2296	

^a^Time of treatment may differ between studies and parasite species.

^b^Selectivity index calculated by taking the ratio of the IC_50_ for the tested mammalian cell line(s) to the IC_50_ for the parasite.

^c^Not determined.

^d^Further tested in in vivo studies and demonstrated promising activity.

Despite the successful examples of natural products for treating apicomplexan parasites, several intrinsic challenges may have contributed to a decline in their consideration as therapeutic agents. Critically important to drug development efforts is the issue of large-scale drug supply. While in vitro efficacy testing can be achieved with milligram quantities of a compound, larger amounts, in the order of grams, are typically needed for downstream studies to comprehensively evaluate compound potential as a viable drug candidate^196^. This becomes a major challenge when the natural products of interest are derived from microbes that cannot be cultured under standard laboratory conditions^197^. Given that over 99% of bacteria from environmental samples are unculturable, discovering and developing entirely new scaffolds with novel mechanisms of action becomes an even greater obstacle^198^. Furthermore, many promising natural products require chemical optimisation to reduce cytotoxicity and improve pharmacokinetic and pharmacodynamic properties, as was the case with quinine, artemisinin, lapachol and febrifugine, among others. While lapachol and febrifugine are relatively small and non-complex, generating structurally diverse analogues of larger and more complex natural products through synthetic chemistry can be a challenging and resource-intensive process that may not be economically realistic, especially for apicomplexan diseases in livestock^199^. Encouragingly, following the example of the synthetic optimisation of erythromycin to azithromycin^200,201^, whose importance is demonstrated by being on the WHO list of essential medicines^78^, successful synthetic optimisation of complex natural products is possible and can enhance their pharmacology, efficacy, safety and cost of production.

Recent advances in synthetic biology may offer new pathways to enhance the feasibility and scalability of natural product drug development. These methods involve introducing and expressing biosynthetic genes from native producers in host organisms that are easier to culture and genetically manipulate, allowing increased yields of valuable and difficult-to-obtain compounds^202,203^. Additionally, attempts to engineer certain enzymes by synthetic biology have demonstrated the potential to generate new natural product derivatives^203,204^, which may be useful in cases where promising natural scaffolds are not feasibly modifiable by synthetic chemistry. However, these methods are still in their early stages and require wider testing to establish utility in the field of natural product drug discovery and development.

## Conclusions

Apicomplexan parasites continue to impose a significant global health and economic burden through widespread diseases affecting both human health and livestock welfare and productivity. Moreover, the emergence of drug resistance to existing treatments poses an additional challenge in managing these infections. Given the historical success of natural products and the abundance of naturally occurring sources still yet to be explored, there remains considerable potential to identify new drug candidates against apicomplexans, both from currently available compound libraries and from novel natural products discovered through innovative identification programs covering a diverse range of plants, fungi, bacteria, and marine organisms^205–207^. However, despite the substantial disease burden of these parasites, the financial incentives for drug development may be limited for many apicomplexan diseases unless the drug has a low cost of production and offers excellent efficacy and safety with a practical dosage regimen to ensure treatment adherence, especially for those that primarily affect low- and middle- income countries. Recent technological advancements in areas like synthetic biology may offer an opportunity to make natural product drug development and production more affordable and feasible for diseases with low research investment. To fully optimise the potential of natural products, discovery and screening efforts should be combined with modern medicinal chemistry to help optimise the efficacy, scalability and development of new drugs, ultimately improving the likelihood of successful market translation. Therefore, revisiting natural products as a source of next-generation drugs remains a valuable pathway forward to address the growing global health and economic burden caused by apicomplexan parasites in humans and livestock.

## Data Availability

No datasets were generated or analysed during the current study.
